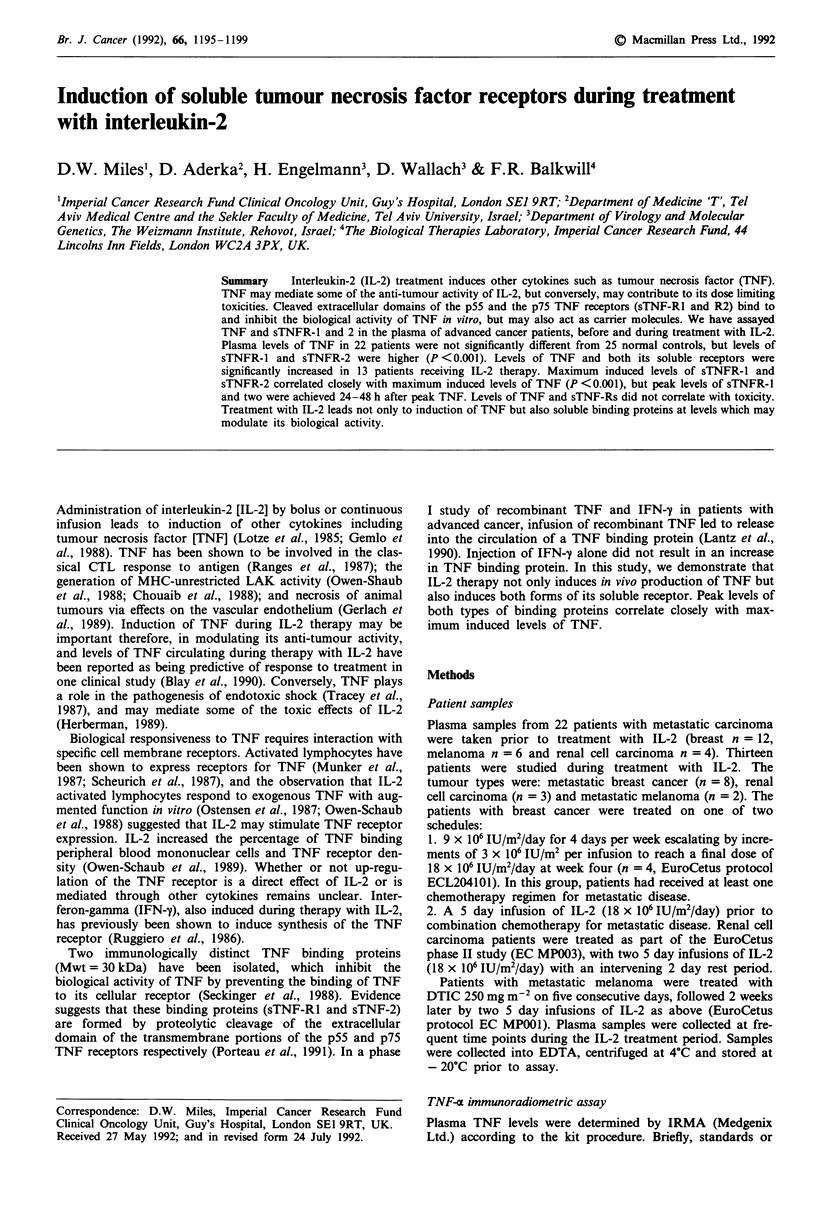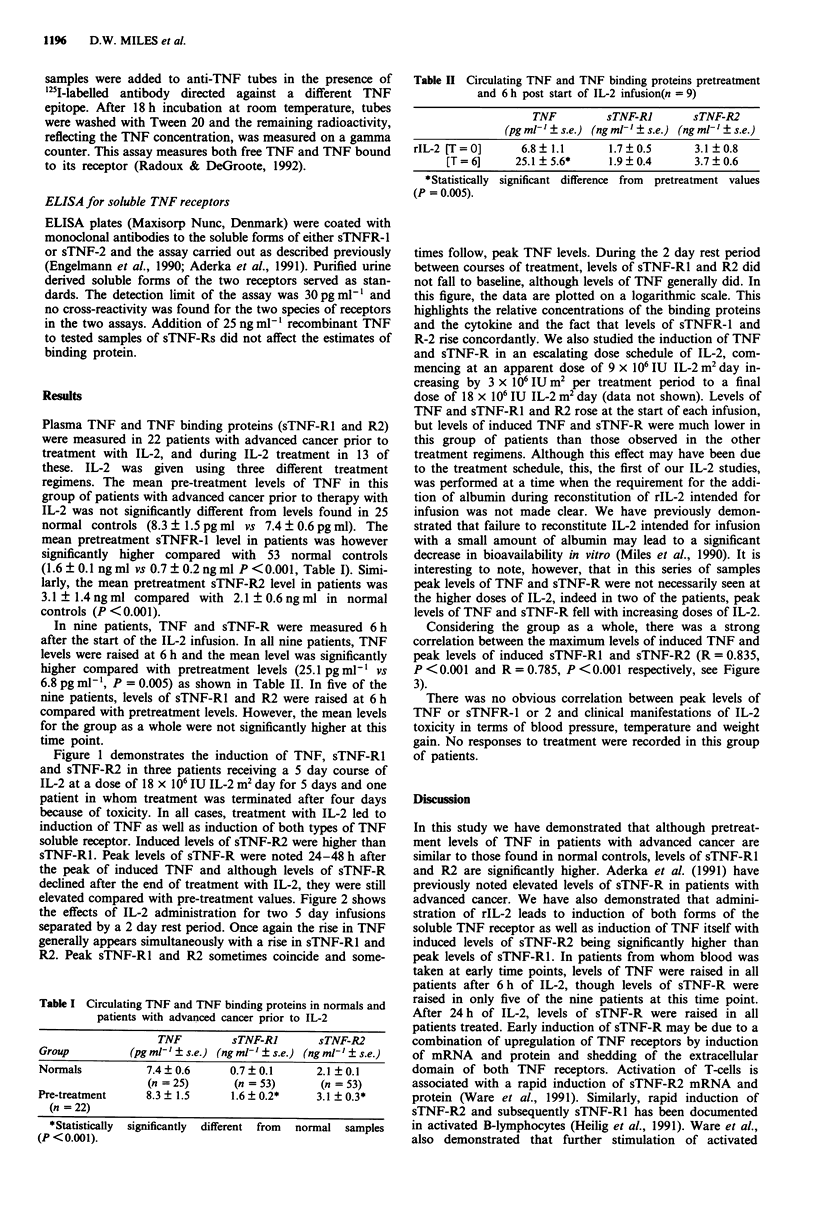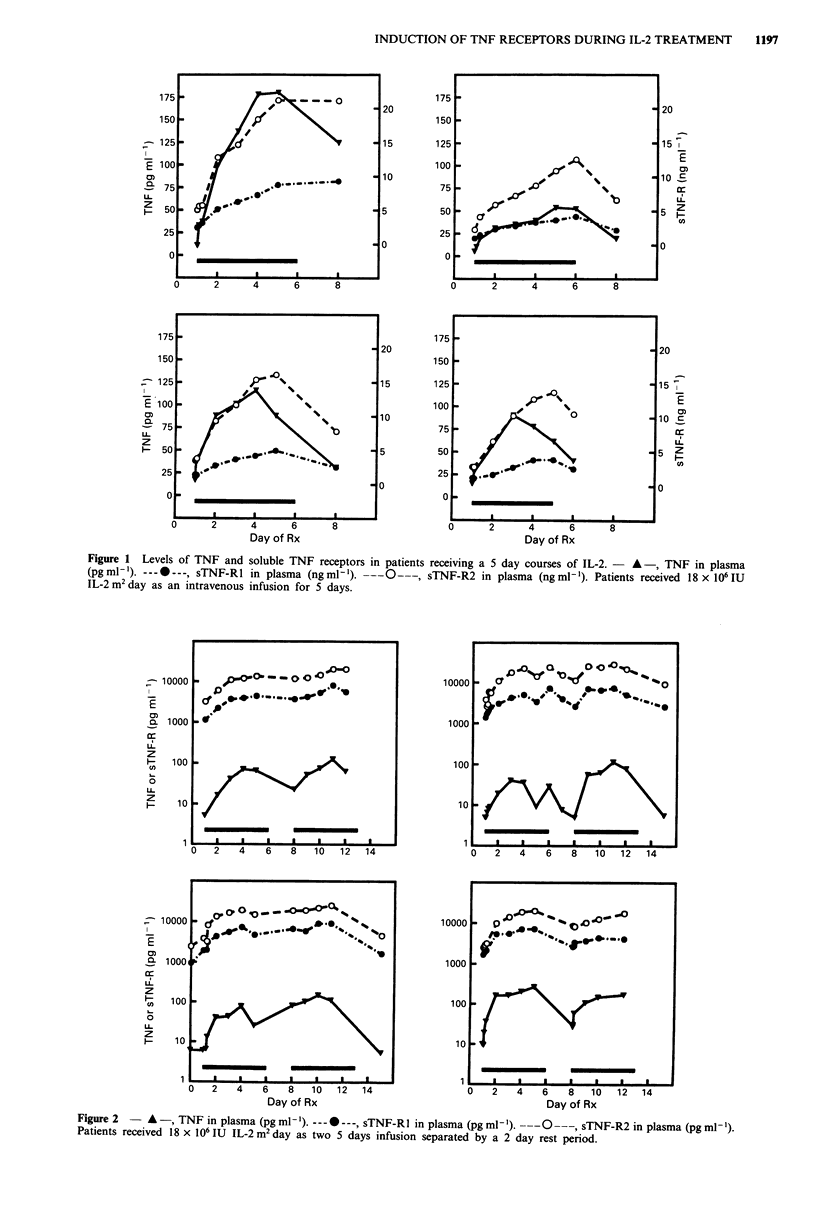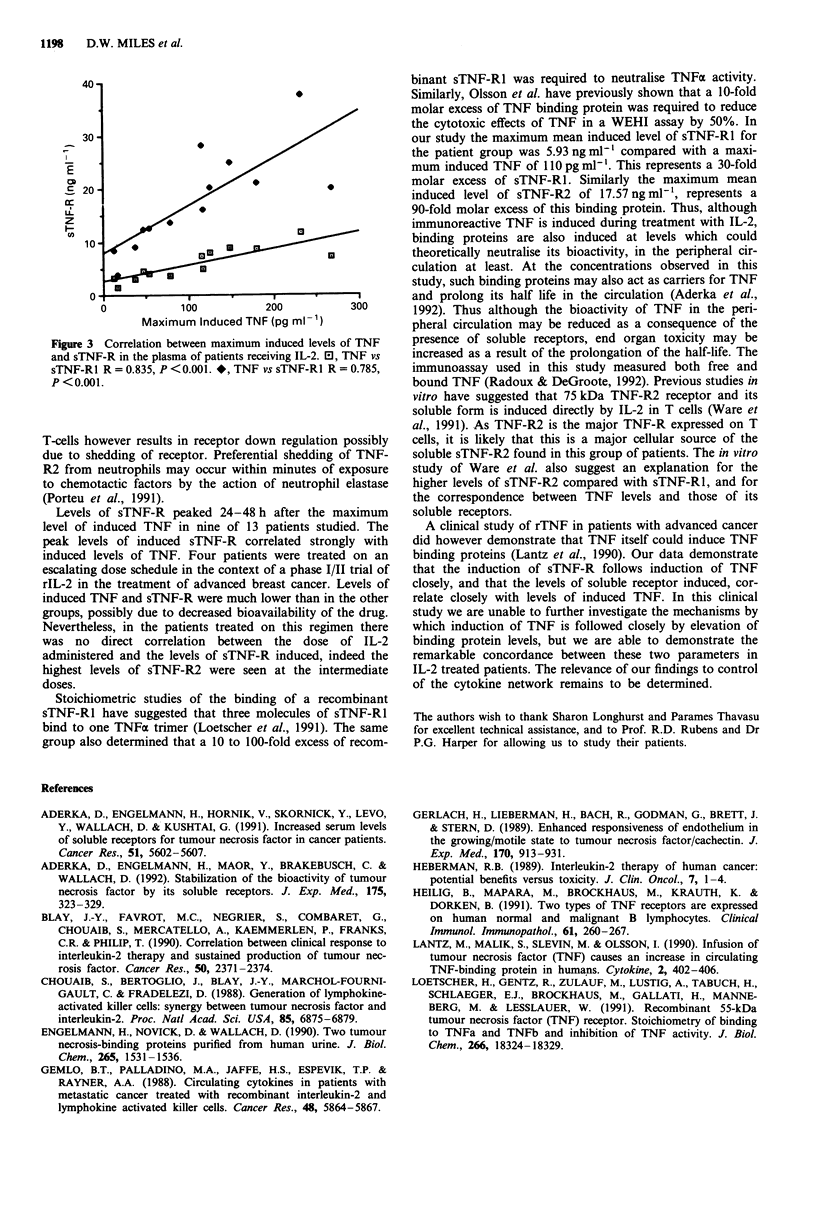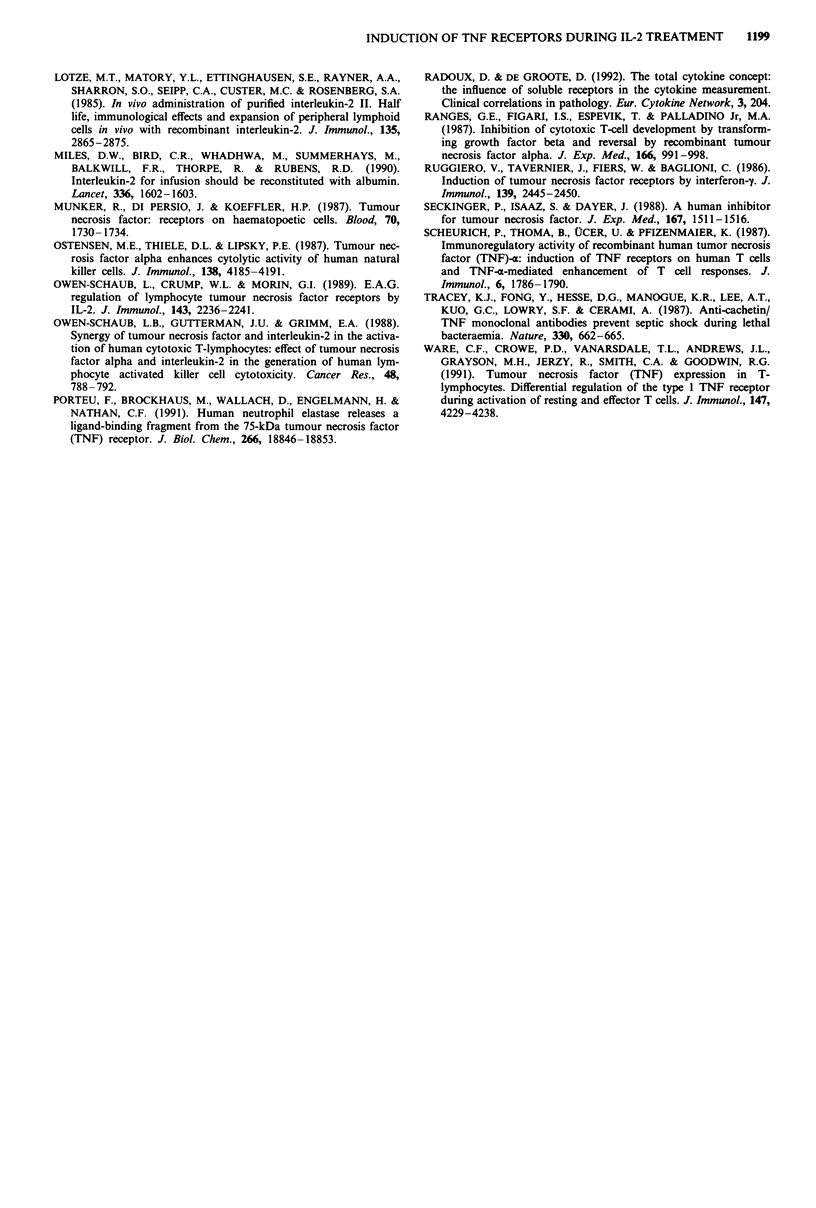# Induction of soluble tumour necrosis factor receptors during treatment with interleukin-2.

**DOI:** 10.1038/bjc.1992.435

**Published:** 1992-12

**Authors:** D. W. Miles, D. Aderka, H. Engelmann, D. Wallach, F. R. Balkwill

**Affiliations:** Imperial Cancer Research Fund Clinical Oncology Unit, Guy's Hospital, London, UK.

## Abstract

Interleukin-2 (IL-2) treatment induces other cytokines such as tumour necrosis factor (TNF) TNF may mediate some of the anti-tumour activity of IL-2, but conversely, may contribute to its dose limiting toxicities. Cleaved extracellular domains of the p55 and the p75 TNF receptors (sTNF-R1 and R2) bind to and inhibit the biological activity of TNF in vitro, but may also act as carrier molecules. We have assayed TNF and sTNFR-1 and 2 in the plasma of advanced cancer patients, before and during treatment with IL-2. Plasma levels of TNF in 22 patients were not significantly different from 25 normal controls, but levels of sTNFR-1 and sTNFR-2 were higher (P < 0.001). Levels of TNF and both its soluble receptors were significantly increased in 13 patients receiving IL-2 therapy. Maximum induced levels of sTNFR-1 and sTNFR-2 correlated closely with maximum induced levels of TNF (P < 0.001), but peak levels of sTNFR-1 and two were achieved 24-48 h after peak TNF. Levels of TNF and sTNF-Rs did not correlate with toxicity. Treatment with IL-2 leads not only to induction of TNF but also soluble binding proteins at levels which may modulate its biological activity.


Br. J. Cancer (1992), 66, 1195–1199 © Macmillan Press Ltd., 1992

### Induction of soluble tumour necrosis factor receptors during treatment with interleukin-2

D.W. Miles[1], D. Aderka[2], H. Engelmann[3], D. Wallach[3] & F.R. Balkwill[4]

[1]*Imperial Cancer Research Fund Clinical Oncology Unit, Guy's Hospital, London SE1 9RT;* [2]*Department of Medicine 'T', Tel Aviv Medical Centre and the Sekler Faculty of Medicine, Tel Aviv University, Israel;* [3]*Department of Virology and Molecular Genetics, The Weizmann Institute, Rehovot, Israel;* [4]*The Biological Therapies Laboratory, Imperial Cancer Research Fund, 44 Lincolns Inn Fields, London WC2A 3PX, UK.*

**Summary** Interleukin-2 (IL-2) treatment induces other cytokines such as tumour necrosis factor (TNF). TNF may mediate some of the anti-tumour activity of IL-2, but conversely, may contribute to its dose limiting toxicities. Cleaved extracellular domains of the p55 and the p75 TNF receptors (sTNF-R1 and R2) bind to and inhibit the biological activity of TNF *in vitro*, but may also act as carrier molecules. We have assayed TNF and sTNFR-1 and 2 in the plasma of advanced cancer patients, before and during treatment with IL-2. Plasma levels of TNF in 22 patients were not significantly different from 25 normal controls, but levels of sTNFR-1 and sTNFR-2 were higher ($P < 0.001$). Levels of TNF and both its soluble receptors were significantly increased in 13 patients receiving IL-2 therapy. Maximum induced levels of sTNFR-1 and sTNFR-2 correlated closely with maximum induced levels of TNF ($P < 0.001$), but peak levels of sTNFR-1 and two were achieved 24–48 h after peak TNF. Levels of TNF and sTNF-Rs did not correlate with toxicity. Treatment with IL-2 leads not only to induction of TNF but also soluble binding proteins at levels which may modulate its biological activity.

Administration of interleukin-2 [IL-2] by bolus or continuous infusion leads to induction of other cytokines including tumour necrosis factor [TNF] (Lotze *et al.*, 1985; Gemlo *et al.*, 1988). TNF has been shown to be involved in the classical CTL response to antigen (Ranges *et al.*, 1987); the generation of MHC-unrestricted LAK activity (Owen-Shaub *et al.*, 1988; Chouaib *et al.*, 1988); and necrosis of animal tumours via effects on the vascular endothelium (Gerlach *et al.*, 1989). Induction of TNF during IL-2 therapy may be important therefore, in modulating its anti-tumour activity, and levels of TNF circulating during therapy with IL-2 have been reported as being predictive of response to treatment in one clinical study (Blay *et al.*, 1990). Conversely, TNF plays a role in the pathogenesis of endotoxic shock (Tracey *et al.*, 1987), and may mediate some of the toxic effects of IL-2 (Herberman, 1989).

Biological responsiveness to TNF requires interaction with specific cell membrane receptors. Activated lymphocytes have been shown to express receptors for TNF (Munker *et al.*, 1987; Scheurich *et al.*, 1987), and the observation that IL-2 activated lymphocytes respond to exogenous TNF with augmented function *in vitro* (Ostensen *et al.*, 1987; Owen-Schaub *et al.*, 1988) suggested that IL-2 may stimulate TNF receptor expression. IL-2 increased the percentage of TNF binding peripheral blood mononuclear cells and TNF receptor density (Owen-Schaub *et al.*, 1989). Whether or not up-regulation of the TNF receptor is a direct effect of IL-2 or is mediated through other cytokines remains unclear. Interferon-gamma (IFN-γ), also induced during therapy with IL-2, has previously been shown to induce synthesis of the TNF receptor (Ruggiero *et al.*, 1986).

Two immunologically distinct TNF binding proteins (Mwt = 30 kDa) have been isolated, which inhibit the biological activity of TNF by preventing the binding of TNF to its cellular receptor (Seckinger *et al.*, 1988). Evidence suggests that these binding proteins (sTNF-R1 and sTNF-2) are formed by proteolytic cleavage of the extracellular domain of the transmembrane portions of the p55 and p75 TNF receptors respectively (Porteau *et al.*, 1991). In a phase I study of recombinant TNF and IFN-γ in patients with advanced cancer, infusion of recombinant TNF led to release into the circulation of a TNF binding protein (Lantz *et al.*, 1990). Injection of IFN-γ alone did not result in an increase in TNF binding protein. In this study, we demonstrate that IL-2 therapy not only induces *in vivo* production of TNF but also induces both forms of its soluble receptor. Peak levels of both types of binding proteins correlate closely with maximum induced levels of TNF.

### Methods

### *Patient samples*

Plasma samples from 22 patients with metastatic carcinoma were taken prior to treatment with IL-2 (breast $n = 12$, melanoma $n = 6$ and renal cell carcinoma $n = 4$). Thirteen patients were studied during treatment with IL-2. The tumour types were: metastatic breast cancer ($n = 8$), renal cell carcinoma ($n = 3$) and metastatic melanoma ($n = 2$). The patients with breast cancer were treated on one of two schedules:

1. $9 \times 10^6$ IU/m$^2$/day for 4 days per week escalating by increments of $3 \times 10^6$ IU/m$^2$ per infusion to reach a final dose of $18 \times 10^6$ IU/m$^2$/day at week four ($n = 4$, EuroCetus protocol ECL204101). In this group, patients had received at least one chemotherapy regimen for metastatic disease.
2. A 5 day infusion of IL-2 ($18 \times 10^6$ IU/m$^2$/day) prior to combination chemotherapy for metastatic disease. Renal cell carcinoma patients were treated as part of the EuroCetus phase II study (EC MP003), with two 5 day infusions of IL-2 ($18 \times 10^6$ IU/m$^2$/day) with an intervening 2 day rest period.

Patients with metastatic melanoma were treated with DTIC 250 mg m$^{-2}$ on five consecutive days, followed 2 weeks later by two 5 day infusions of IL-2 as above (EuroCetus protocol EC MP001). Plasma samples were collected at frequent time points during the IL-2 treatment period. Samples were collected into EDTA, centrifuged at 4°C and stored at – 20°C prior to assay.

### *TNF-α immunoradiometric assay*

Plasma TNF levels were determined by IRMA (Medgenix Ltd.) according to the kit procedure. Briefly, standards or

Correspondence: D.W. Miles, Imperial Cancer Research Fund Clinical Oncology Unit, Guy's Hospital, London SE1 9RT, UK.
Received 27 May 1992; and in revised form 24 July 1992.

samples were added to anti-TNF tubes in the presence of $^{125}$I-labelled antibody directed against a different TNF epitope. After 18 h incubation at room temperature, tubes were washed with Tween 20 and the remaining radioactivity, reflecting the TNF concentration, was measured on a gamma counter. This assay measures both free TNF and TNF bound to its receptor (Radoux & DeGroote, 1992).

*ELISA for soluble TNF receptors*

ELISA plates (Maxisorp Nunc, Denmark) were coated with monoclonal antibodies to the soluble forms of either sTNFR-1 or sTNF-2 and the assay carried out as described previously (Engelmann *et al.*, 1990; Aderka *et al.*, 1991). Purified urine derived soluble forms of the two receptors served as standards. The detection limit of the assay was 30 pg ml$^{-1}$ and no cross-reactivity was found for the two species of receptors in the two assays. Addition of 25 ng ml$^{-1}$ recombinant TNF to tested samples of sTNF-Rs did not affect the estimates of binding protein.

### Results

Plasma TNF and TNF binding proteins (sTNF-R1 and R2) were measured in 22 patients with advanced cancer prior to treatment with IL-2, and during IL-2 treatment in 13 of these. IL-2 was given using three different treatment regimens. The mean pre-treatment levels of TNF in this group of patients with advanced cancer prior to therapy with IL-2 was not significantly different from levels found in 25 normal controls (8.3 ± 1.5 pg ml *vs* 7.4 ± 0.6 pg ml). The mean pretreatment sTNFR-1 level in patients was however significantly higher compared with 53 normal controls (1.6 ± 0.1 ng ml *vs* 0.7 ± 0.2 ng ml $P < 0.001$, Table I). Similarly, the mean pretreatment sTNF-R2 level in patients was 3.1 ± 1.4 ng ml compared with 2.1 ± 0.6 ng ml in normal controls ($P < 0.001$).

In nine patients, TNF and sTNF-R were measured 6 h after the start of the IL-2 infusion. In all nine patients, TNF levels were raised at 6 h and the mean level was significantly higher compared with pretreatment levels (25.1 pg ml$^{-1}$ *vs* 6.8 pg ml$^{-1}$, $P = 0.005$) as shown in Table II. In five of the nine patients, levels of sTNF-R1 and R2 were raised at 6 h compared with pretreatment levels. However, the mean levels for the group as a whole were not significantly higher at this time point.

Figure 1 demonstrates the induction of TNF, sTNF-R1 and sTNF-R2 in three patients receiving a 5 day course of IL-2 at a dose of 18 × 10$^6$ IU IL-2 m$^2$ day for 5 days and one patient in whom treatment was terminated after four days because of toxicity. In all cases, treatment with IL-2 led to induction of TNF as well as induction of both types of TNF soluble receptor. Induced levels of sTNF-R2 were higher than sTNF-R1. Peak levels of sTNF-R were noted 24–48 h after the peak of induced TNF and although levels of sTNF-R declined after the end of treatment with IL-2, they were still elevated compared with pre-treatment values. Figure 2 shows the effects of IL-2 administration for two 5 day infusions separated by a 2 day rest period. Once again the rise in TNF generally appears simultaneously with a rise in sTNF-R1 and R2. Peak sTNF-R1 and R2 sometimes coincide and sometimes follow, peak TNF levels. During the 2 day rest period between courses of treatment, levels of sTNF-R1 and R2 did not fall to baseline, although levels of TNF generally did. In this figure, the data are plotted on a logarithmic scale. This highlights the relative concentrations of the binding proteins and the cytokine and the fact that levels of sTNFR-1 and R-2 rise concordantly. We also studied the induction of TNF and sTNF-R in an escalating dose schedule of IL-2, commencing at an apparent dose of 9 × 10$^6$ IU IL-2 m$^2$ day increasing by 3 × 10$^6$ IU m$^2$ per treatment period to a final dose of 18 × 10$^6$ IU IL-2 m$^2$ day (data not shown). Levels of TNF and sTNF-R1 and R2 rose at the start of each infusion, but levels of induced TNF and sTNF-R were much lower in this group of patients than those observed in the other treatment regimens. Although this effect may have been due to the treatment schedule, this, the first of our IL-2 studies, was performed at a time when the requirement for the addition of albumin during reconstitution of rIL-2 intended for infusion was not made clear. We have previously demonstrated that failure to reconstitute IL-2 intended for infusion with a small amount of albumin may lead to a significant decrease in bioavailability *in vitro* (Miles *et al.*, 1990). It is interesting to note, however, that in this series of samples peak levels of TNF and sTNF-R were not necessarily seen at the higher doses of IL-2, indeed in two of the patients, peak levels of TNF and sTNF-R fell with increasing doses of IL-2.

Considering the group as a whole, there was a strong correlation between the maximum levels of induced TNF and peak levels of induced sTNF-R1 and sTNF-R2 (R = 0.835, $P < 0.001$ and R = 0.785, $P < 0.001$ respectively, see Figure 3).

There was no obvious correlation between peak levels of TNF or sTNFR-1 or 2 and clinical manifestations of IL-2 toxicity in terms of blood pressure, temperature and weight gain. No responses to treatment were recorded in this group of patients.

**Table I** Circulating TNF and TNF binding proteins in normals and patients with advanced cancer prior to IL-2

| Group | TNF (pg ml$^{-1}$ ± s.e.) | sTNF-R1 (ng ml$^{-1}$ ± s.e.) | sTNF-R2 (ng ml$^{-1}$ ± s.e.) |
|---|---|---|---|
| Normals | 7.4 ± 0.6 ($n$ = 25) | 0.7 ± 0.1 ($n$ = 53) | 2.1 ± 0.1 ($n$ = 53) |
| Pre-treatment ($n$ = 22) | 8.3 ± 1.5 | 1.6 ± 0.2* | 3.1 ± 0.3* |

*Statistically significantly different from normal samples ($P < 0.001$).

**Table II** Circulating TNF and TNF binding proteins pretreatment and 6 h post start of IL-2 infusion($n$ = 9)

| | TNF (pg ml$^{-1}$ ± s.e.) | sTNF-R1 (ng ml$^{-1}$ ± s.e.) | sTNF-R2 (ng ml$^{-1}$ ± s.e.) |
|---|---|---|---|
| rIL-2 [T = 0] | 6.8 ± 1.1 | 1.7 ± 0.5 | 3.1 ± 0.8 |
| [T = 6] | 25.1 ± 5.6* | 1.9 ± 0.4 | 3.7 ± 0.6 |

*Statistically significant difference from pretreatment values ($P = 0.005$).

### Discussion

In this study we have demonstrated that although pretreatment levels of TNF in patients with advanced cancer are similar to those found in normal controls, levels of sTNF-R1 and R2 are significantly higher. Aderka *et al.* (1991) have previously noted elevated levels of sTNF-R in patients with advanced cancer. We have also demonstrated that administration of rIL-2 leads to induction of both forms of the soluble TNF receptor as well as induction of TNF itself with induced levels of sTNF-R2 being significantly higher than peak levels of sTNF-R1. In patients from whom blood was taken at early time points, levels of TNF were raised in all patients after 6 h of IL-2, though levels of sTNF-R were raised in only five of the nine patients at this time point. After 24 h of IL-2, levels of sTNF-R were raised in all patients treated. Early induction of sTNF-R may be due to a combination of upregulation of TNF receptors by induction of mRNA and protein and shedding of the extracellular domain of both TNF receptors. Activation of T-cells is associated with a rapid induction of sTNF-R2 mRNA and protein (Ware *et al.*, 1991). Similarly, rapid induction of sTNF-R2 and subsequently sTNF-R1 has been documented in activated B-lymphocytes (Heilig *et al.*, 1991). Ware *et al.*, also demonstrated that further stimulation of activated

**Figure 1** Levels of TNF and soluble TNF receptors in patients receiving a 5 day courses of IL-2. — ▲—, TNF in plasma (pg ml$^{-1}$). ---●---, sTNF-R1 in plasma (ng ml$^{-1}$). ---O---, sTNF-R2 in plasma (ng ml$^{-1}$). Patients received $18 \times 10^6$ IU IL-2 m$^2$ day as an intravenous infusion for 5 days.

**Figure 2** — ▲—, TNF in plasma (pg ml$^{-1}$). ---●---, sTNF-R1 in plasma (pg ml$^{-1}$). ---O---, sTNF-R2 in plasma (pg ml$^{-1}$). Patients received $18 \times 10^6$ IU IL-2 m$^2$ day as two 5 days infusion separated by a 2 day rest period.

**Figure 3** Correlation between maximum induced levels of TNF and sTNF-R in the plasma of patients receiving IL-2. ☐, TNF *vs* sTNF-R1 R = 0.835, $P < 0.001$. ◆, TNF *vs* sTNF-R1 R = 0.785, $P < 0.001$.

T-cells however results in receptor down regulation possibly due to shedding of receptor. Preferential shedding of TNF-R2 from neutrophils may occur within minutes of exposure to chemotactic factors by the action of neutrophil elastase (Porteu *et al.*, 1991).

Levels of sTNF-R peaked 24–48 h after the maximum level of induced TNF in nine of 13 patients studied. The peak levels of induced sTNF-R correlated strongly with induced levels of TNF. Four patients were treated on an escalating dose schedule in the context of a phase I/II trial of rIL-2 in the treatment of advanced breast cancer. Levels of induced TNF and sTNF-R were much lower than in the other groups, possibly due to decreased bioavailability of the drug. Nevertheless, in the patients treated on this regimen there was no direct correlation between the dose of IL-2 administered and the levels of sTNF-R induced, indeed the highest levels of sTNF-R2 were seen at the intermediate doses.

Stoichiometric studies of the binding of a recombinant sTNF-R1 have suggested that three molecules of sTNF-R1 bind to one TNFα trimer (Loetscher *et al.*, 1991). The same group also determined that a 10 to 100-fold excess of recombinant sTNF-R1 was required to neutralise TNFα activity. Similarly, Olsson *et al.* have previously shown that a 10-fold molar excess of TNF binding protein was required to reduce the cytotoxic effects of TNF in a WEHI assay by 50%. In our study the maximum mean induced level of sTNF-R1 for the patient group was 5.93 ng ml$^{-1}$ compared with a maximum induced TNF of 110 pg ml$^{-1}$. This represents a 30-fold molar excess of sTNF-R1. Similarly the maximum mean induced level of sTNF-R2 of 17.57 ng ml$^{-1}$, represents a 90-fold molar excess of this binding protein. Thus, although immunoreactive TNF is induced during treatment with IL-2, binding proteins are also induced at levels which could theoretically neutralise its bioactivity, in the peripheral circulation at least. At the concentrations observed in this study, such binding proteins may also act as carriers for TNF and prolong its half life in the circulation (Aderka *et al.*, 1992). Thus although the bioactivity of TNF in the peripheral circulation may be reduced as a consequence of the presence of soluble receptors, end organ toxicity may be increased as a result of the prolongation of the half-life. The immunoassay used in this study measured both free and bound TNF (Radoux & DeGroote, 1992). Previous studies *in vitro* have suggested that 75 kDa TNF-R2 receptor and its soluble form is induced directly by IL-2 in T cells (Ware *et al.*, 1991). As TNF-R2 is the major TNF-R expressed on T cells, it is likely that this is a major cellular source of the soluble sTNF-R2 found in this group of patients. The *in vitro* study of Ware *et al.* also suggest an explanation for the higher levels of sTNF-R2 compared with sTNF-R1, and for the correspondence between TNF levels and those of its soluble receptors.

A clinical study of rTNF in patients with advanced cancer did however demonstrate that TNF itself could induce TNF binding proteins (Lantz *et al.*, 1990). Our data demonstrate that the induction of sTNF-R follows induction of TNF closely, and that the levels of soluble receptor induced, correlate closely with levels of induced TNF. In this clinical study we are unable to further investigate the mechanisms by which induction of TNF is followed closely by elevation of binding protein levels, but we are able to demonstrate the remarkable concordance between these two parameters in IL-2 treated patients. The relevance of our findings to control of the cytokine network remains to be determined.

The authors wish to thank Sharon Longhurst and Parames Thavasu for excellent technical assistance, and to Prof. R.D. Rubens and Dr P.G. Harper for allowing us to study their patients.

### References

ADERKA, D., ENGELMANN, H., HORNIK, V., SKORNICK, Y., LEVO, Y., WALLACH, D. & KUSHTAI, G. (1991). Increased serum levels of soluble receptors for tumour necrosis factor in cancer patients. *Cancer Res.*, **51**, 5602–5607.

ADERKA, D., ENGELMANN, H., MAOR, Y., BRAKEBUSCH, C. & WALLACH, D. (1992). Stabilization of the bioactivity of tumour necrosis factor by its soluble receptors. *J. Exp. Med.*, **175**, 323–329.

BLAY, J.-Y., FAVROT, M.C., NEGRIER, S., COMBARET, G., CHOUAIB, S., MERCATELLO, A., KAEMMERLEN, P., FRANKS, C.R. & PHILIP, T. (1990). Correlation between clinical response to interleukin-2 therapy and sustained production of tumour necrosis factor. *Cancer Res.*, **50**, 2371–2374.

CHOUAIB, S., BERTOGLIO, J., BLAY, J.-Y., MARCHOL-FOURNIGAULT, C. & FRADELEZI, D. (1988). Generation of lymphokine-activated killer cells: synergy between tumour necrosis factor and interleukin-2. *Proc. Natl Acad. Sci. USA*, **85**, 6875–6879.

ENGELMANN, H., NOVICK, D. & WALLACH, D. (1990). Two tumour necrosis-binding proteins purified from human urine. *J. Biol. Chem.*, **265**, 1531–1536.

GEMLO, B.T., PALLADINO, M.A., JAFFE, H.S., ESPEVIK, T.P. & RAYNER, A.A. (1988). Circulating cytokines in patients with metastatic cancer treated with recombinant interleukin-2 and lymphokine activated killer cells. *Cancer Res.*, **48**, 5864–5867.

GERLACH, H., LIEBERMAN, H., BACH, R., GODMAN, G., BRETT, J. & STERN, D. (1989). Enhanced responsiveness of endothelium in the growing/motile state to tumour necrosis factor/cachectin. *J. Exp. Med.*, **170**, 913–931.

HEBERMAN, R.B. (1989). Interleukin-2 therapy of human cancer: potential benefits versus toxicity. *J. Clin. Oncol.*, **7**, 1–4.

HEILIG, B., MAPARA, M., BROCKHAUS, M., KRAUTH, K. & DORKEN, B. (1991). Two types of TNF receptors are expressed on human normal and malignant B lymphocytes. *Clinical Immunol. Immunopathol.*, **61**, 260–267.

LANTZ, M., MALIK, S., SLEVIN, M. & OLSSON, I. (1990). Infusion of tumour necrosis factor (TNF) causes an increase in circulating TNF-binding protein in humans. *Cytokine*, **2**, 402–406.

LOETSCHER, H., GENTZ, R., ZULAUF, M., LUSTIG, A., TABUCH, H., SCHLAEGER, E.J., BROCKHAUS, M., GALLATI, H., MANNEBERG, M. & LESSLAUER, W. (1991). Recombinant 55-kDa tumour necrosis factor (TNF) receptor. Stoichiometry of binding to TNFa and TNFb and inhibition of TNF activity. *J. Biol. Chem.*, **266**, 18324–18329.

LOTZE, M.T., MATORY, Y.L., ETTINGHAUSEN, S.E., RAYNER, A.A., SHARRON, S.O., SEIPP, C.A., CUSTER, M.C. & ROSENBERG, S.A. (1985). *In vivo* administration of purified interleukin-2 II. Half life, immunological effects and expansion of peripheral lymphoid cells *in vivo* with recombinant interleukin-2. *J. Immunol.*, **135**, 2865–2875.

MILES, D.W., BIRD, C.R., WHADHWA, M., SUMMERHAYS, M., BALKWILL, F.R., THORPE, R. & RUBENS, R.D. (1990). Interleukin-2 for infusion should be reconstituted with albumin. *Lancet*, **336**, 1602–1603.

MUNKER, R., DI PERSIO, J. & KOEFFLER, H.P. (1987). Tumour necrosis factor: receptors on haematopoetic cells. *Blood*, **70**, 1730–1734.

OSTENSEN, M.E., THIELE, D.L. & LIPSKY, P.E. (1987). Tumour necrosis factor alpha enhances cytolytic activity of human natural killer cells. *J. Immunol.*, **138**, 4185–4191.

OWEN-SCHAUB, L., CRUMP, W.L. & MORIN, G.I. (1989). E.A.G. regulation of lymphocyte tumour necrosis factor receptors by IL-2. *J. Immunol.*, **143**, 2236–2241.

OWEN-SCHAUB, L.B., GUTTERMAN, J.U. & GRIMM, E.A. (1988). Synergy of tumour necrosis factor and interleukin-2 in the activation of human cytotoxic T-lymphocytes: effect of tumour necrosis factor alpha and interleukin-2 in the generation of human lymphocyte activated killer cell cytotoxicity. *Cancer Res.*, **48**, 788–792.

PORTEU, F., BROCKHAUS, M., WALLACH, D., ENGELMANN, H. & NATHAN, C.F. (1991). Human neutrophil elastase releases a ligand-binding fragment from the 75-kDa tumour necrosis factor (TNF) receptor. *J. Biol. Chem.*, **266**, 18846–18853.

RADOUX, D. & DE GROOTE, D. (1992). The total cytokine concept: the influence of soluble receptors in the cytokine measurement. Clinical correlations in pathology. *Eur. Cytokine Network*, **3**, 204.

RANGES, G.E., FIGARI, I.S., ESPEVIK, T. & PALLADINO Jr, M.A. (1987). Inhibition of cytotoxic T-cell development by transforming growth factor beta and reversal by recombinant tumour necrosis factor alpha. *J. Exp. Med.*, **166**, 991–998.

RUGGIERO, V., TAVERNIER, J., FIERS, W. & BAGLIONI, C. (1986). Induction of tumour necrosis factor receptors by interferon-γ. *J. Immunol.*, **139**, 2445–2450.

SECKINGER, P., ISAAZ, S. & DAYER, J. (1988). A human inhibitor for tumour necrosis factor. *J. Exp. Med.*, **167**, 1511–1516.

SCHEURICH, P., THOMA, B., ÜCER, U. & PFIZENMAIER, K. (1987). Immunoregulatory activity of recombinant human tumor necrosis factor (TNF)-α: induction of TNF receptors on human T cells and TNF-α-mediated enhancement of T cell responses. *J. Immunol.*, **6**, 1786–1790.

TRACEY, K.J., FONG, Y., HESSE, D.G., MANOGUE, K.R., LEE, A.T., KUO, G.C., LOWRY, S.F. & CERAMI, A. (1987). Anti-cachetin/TNF monoclonal antibodies prevent septic shock during lethal bacteraemia. *Nature*, **330**, 662–665.

WARE, C.F., CROWE, P.D., VANARSDALE, T.L., ANDREWS, J.L., GRAYSON, M.H., JERZY, R., SMITH, C.A. & GOODWIN, R.G. (1991). Tumour necrosis factor (TNF) expression in T-lymphocytes. Differential regulation of the type 1 TNF receptor during activation of resting and effector T cells. *J. Immunol.*, **147**, 4229–4238.